# The Fingerprints of Resonant Frequency for Atomic Vacancy Defect Identification in Graphene

**DOI:** 10.3390/nano11123451

**Published:** 2021-12-20

**Authors:** Liu Chu, Jiajia Shi, Eduardo Souza de Cursi

**Affiliations:** 1 School of Transportation and Civil Engineering, Nantong University, Nantong 226019, China; chuliu@ntu.edu.cn; 2 Département Mécanique, Institut National des Sciences Appliquées de Rouen, 76800 Rouen, France; souza@insa-rouen.fr

**Keywords:** fingerprints, atomic vacancy defects, resonant frequencies, graphene

## Abstract

The identification of atomic vacancy defects in graphene is an important and challenging issue, which involves inhomogeneous spatial randomness and requires high experimental conditions. In this paper, the fingerprints of resonant frequency for atomic vacancy defect identification are provided, based on the database of massive samples. Every possible atomic vacancy defect in the graphene lattice is considered and computed by the finite element model in sequence. Based on the sample database, the histograms of resonant frequency are provided to compare the probability density distributions and interval ranges. Furthermore, the implicit relationship between the locations of the atomic vacancy defects and the resonant frequencies of graphene is established. The fingerprint patterns are depicted by mapping the locations of atomic vacancy defects to the resonant frequency magnitudes. The geometrical characteristics of computed fingerprints are discussed to explore the feasibility of atomic vacancy defects identification. The work in this paper provides meaningful supplementary information for non-destructive defect detection and identification in nanomaterials.

## 1. Introduction

Defects are recognized as the unpredictable and stochastic existence in the lattice of graphene, which seriously compromises the expected performances and properties [1,2]. However, defects also play positive roles by intentionally tailoring the chemical and physical properties, which present promising potentials in a wide range of applications, such as catalysis [3,4], hydrogen storage [5,6], batteries [7,8], and sensors [9,10]. With the current experimental conditions and manufacturing technologies, defects in graphene are unavoidable during growth and production. Generally, the atomic vacancy defects are stochastically distributed in the periodic hexagonal lattice with inhomogeneous spatial randomness. The identifications of defect location, size, density, and track of the dynamic expansion are challenging issues in both the experimental and theoretical aspects.

At present, it is hard to precisely identify the atomic vacancy defects in graphene by the available experimental instruments. Even atomic force microscopes provide satisfactory three-dimensional imaging of defects in local spaces, but the detection of specific locations on the global scale has low efficiency. On the other hand, scanning tunnelling microscopes and transmission electron microscopes are more competitive in the global feature observation, but the resolution for local defects needs improvement. Moreover, defects are unsteady local structure and easily affected by the disturbances of light, heat, or electromagnetic waves [11], which leads to dynamic expansion, geometric transformation, and energy fluctuation in graphene [12]. Furthermore, the random location of defects makes the observation of certain specimens not comparable. However, testing all the possible situations and preparing every special sample is expensive and unrealistic. Therefore, the explorations for defect identification in graphene needs more attention.

In order to develop efficient methods for defects identification in graphene, many analytical and numerical approaches are attempted, such as molecular dynamics (MD) simulation [13], density functional theory [14], continuum elasticity theory [15], and Monte Carlo-based finite element methods [16]. In order to compare the measured results in the experiments, the thermal vibration and resonant frequencies of graphene are the research focus. The observation of thermal vibration is a non-destructive test, with all edges of the graphene sheet clamped by a scanning force microscope or interferometry. However, the identification of the vibration topography of graphene is complex and computationally expensive. Even though emerging machine learning approaches offer solutions for learning patterns from complex data and have been extensively applied in graphene defect investigation [17,18,19], the intrinsic periodic characteristics and symmetrical patterns in the hexagonal lattice for defects identification are not yet completely analyzed and understood.

The fingerprints of graphene are imaged by vibrational sum-frequency spectroscopy and Raman spectroscopy, etc. For example, vibrational fingerprints of residual polymer on transferred CVD graphene are provided by the use of vibrational sum-frequency spectroscopy [20]. The Raman fingerprint is used to probe the number of layers and conduct a quality assessment of mechanically exfoliated graphene [21]. Moreover, Raman fingerprints of graphene, produced by anodic electrochemical exfoliation, are also presented [22]. The position-dependent Fermi velocity fingerprints are figured out based on scanning tunnelling spectra of strained graphene [23]. Furthermore, the harmonic fingerprint of unconventional superconductivity in twisted bilayer graphene is collected [24]. For more physical characteristics of graphene, the spin–orbit interaction fingerprints of a ballistic graphene Josephson junction [25], the enhancing mid-infrared molecular fingerprints by acoustic graphene plasma polarities [26], and the infrared fingerprints of natural 2D talc and plasma phonon coupling in graphene–talc heterostructures [27] are all studied. Therefore, the fingerprints of resonant frequency for vacancy defects identification at the atomic scale is an essential and promising topic.

In this study, the fingerprints of resonant frequency for atomic vacancy defect identification in graphene are depicted, based on finite element computation. The geometrical configuration of graphene lattice and the atomic vacancy defects are introduced in Section 2. Modal analysis, based on the finite element method and the flowchart of the fingerprint compiler, is explained. The probability density distributions of resonant frequencies are provided and discussed in Section 3. Furthermore, the geometrical characteristics of fingerprint patterns are studied to analyze the feasibility of atomic vacancy defect identification. The short conclusion is presented in the last section.

## 2. Materials and Methods

### 2.1. Atomic Vacancy Defects in Graphene

**Figure 1:**
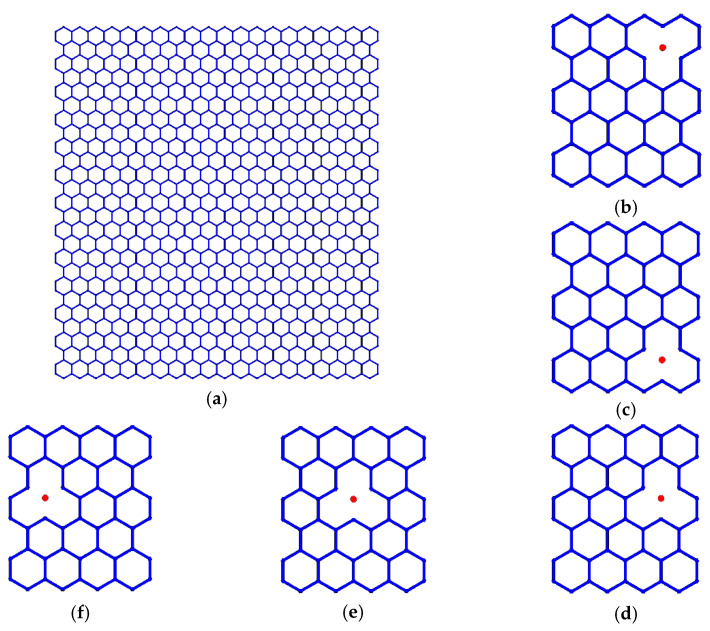
Schematic for graphene honeycomb lattice and examples of atomic vacancy defects ((**a**) presents the periodic honeycomb lattice of pristine graphene, (**b**–**d**) provide the examples of atomic vacancy defects in the *y*-axis direction, (**d**–**f**) present the atomic vacancy defects located in the *x*-axis direction).

The characteristic honeycomb lattice of graphene consists of the periodic hexagons of carbon–carbon covalent bonds, as presented in Figure 1a. In the geometrical configuration, the carbon–carbon covalent bonds are simplified as a beam, and there are 4212 carbon atoms taken into consideration. The geometrical parameters involving the length of carbon covalent bonds and the diameter of the cross-section of the beam (thickness of graphene) are 0.27 nm and 0.032 nm, respectively. The material properties, namely Young’s modulus, Poisson’s ratio, and mass density, are settled as 1.2 TPa, 0.2, and 2700 Kg/m^3^, respectively, according to the reported literature [7,28,29,30].

In addition, the atomic vacancy defects are introduced in sequence in the pristine perfect graphene samples. The locations of the atomic vacancy defects are along the *x*-axis direction (from Figure 1d–f) and the *y*-axis direction (from Figure 1b–d). Each carbon atom in the graphene lattice is compiled with a corresponding number carrying important location information. Recording the numbers of vacancy defected atoms makes it possible to track the exact location in graphene lattice.

### 2.2. Modal Analysis in Finite Element Model

Under the environment of ANSYS parameter design language (Version 14.5, APDL), 6226 elements, 4212 points, and 16,664 nodes are created for the pristine graphene without any vacancy defect. Beam 188 is selected as the effective finite element to mesh the graphene lattice. On the one hand, it is feasible to define the thickness of graphene as the diameter of the cross-section of Beam 188. On the other hand, Beam 188 is the highly efficient element for finite element computation of the resonant frequencies. The equation that governs flexural vibrations of Beam 188 with constant cross-section can be expressed as [28] follows:

(1) $$\frac{EI}{\rho A}\frac{\partial^{4}\xi }{\partial z^{4}}\;-\frac{I}{A}\left(1+\frac{E}{kG}\right)\frac{\partial^{4}\xi }{\partial z^{2}\partial t^{2}\;}+\frac{\partial^{2}\xi }{\partial t^{2}\;}+\frac{\rho I}{kGA}\frac{\partial^{4}\xi }{\partial z^{4}}=0$$

where ξ=ξ(z,t) is the transversal displacement along the *x*-axis at point *z* and time *t*, and *E*, *G*, *ρ* are Young’s modulus, shear modulus, and physical density, respectively. I is the inertia moment and *A* is the area of cross-section. In a normal mode, ξ(z,t) varies harmonically with time as follows:

(2) ξ=[Acos(ωt)+Bsin(ωt)]χ(z)=Csin(ωt+φ)χ(z)

where *A*, *B*, *C*, and φ are the corresponding constants to be determined. ω=2πf  is the angular frequency and χ(z) is a function that determines the normal mode amplitude. Substituting this form of ξ into Equation (1), as follows:

(3) $$\frac{\partial^{4}\chi }{\partial z^{4}}+\frac{\rho \omega^{2}}{M_{r}}\frac{\partial^{2}\chi }{\partial z^{2}}+\frac{\omega^{2}\rho^{2}}{kGE}\left[\omega^{2}-\omega_{c}^{2}\right]\chi =0$$

with $\omega_{c}=2\pi f_{c}=\sqrt{\frac{kGA}{\rho I}}$, where $f_{c}$ is the critical frequency and $\frac{1}{M_{r}}=\frac{1}{E}+\frac{1}{kG}$ is the reduced modulus.

The general solution can be written as follows:

(4) $$\{\begin{array}{l} \chi \left(z\right)=A_{1}\sin\left(K_{1}z\right)+B_{1}\cos\left(K_{1}z\right)+C_{1}e^{K_{2}z}+D_{1}e^{-K_{2}z}\;\;\;\;\;\;\;\;\;\;\;\;\;\;\;\;\;\;\;\;\;\;\;\;\;\omega \leq \omega_{c}\\ \chi \left(z\right)=A_{2}\sin\left(K_{1}z\right)+B_{2}\cos\left(K_{1}z\right)+C_{2}\sin\left(K_{2}z\right)+D_{2}\cos\left(K_{2}z\right)\;\;\;\;\;\;\;\;\omega >\omega_{c} \end{array}$$

The coefficients *A_i_*, *B_i_*, *C_i_*, and *D_i_* are different from zero, and the solutions of the equations include functions depending on both *K*_1_ and *K*_2_.

(5) $$\{\begin{array}{l} K_{1}=\sqrt{\frac{\rho \omega^{2}}{2M_{r}}+\sqrt{{\left(\frac{\rho \omega^{2}}{2M_{r}}\right)}^{2}-\frac{\rho \omega^{2}}{kGE}\left(\omega^{2}-\omega_{c}^{2}\right)}}\\ K_{2}=\sqrt{S\left[\frac{\rho \omega^{2}}{2M_{r}}-\sqrt{{\left(\frac{\rho \omega^{2}}{2M_{r}}\right)}^{2}-\frac{\rho \omega^{2}}{kGE}\left(\omega^{2}-\omega_{c}^{2}\right)}\right]} \end{array}\;\;\;\;\;\;\;\;\;\;\;With\;\;S=\{\begin{array}{c} 1\;\;\;\;if\;\omega \leq \omega_{c}\;\\ -1\;\;\;\;\omega >\omega_{c} \end{array}$$

For free vibration analysis, based on the principle of virtual work, the weak form of the equation is simplified as follows:

(6) $$\int_{0}^{L}EI\frac{\partial \theta }{\partial x}\delta \left(\frac{\partial \theta }{\partial x}\right)dx+\int_{0}^{L}kGA\left(\frac{\partial \xi }{\partial x}-\theta \right)\delta \left(\frac{\partial \xi }{\partial x}-\theta \right)dx=\int_{0}^{L}\delta \xi \rho A\ddot{\xi }dx+\int_{0}^{L}\delta \theta \rho I\ddot{\theta }dx$$

As defined above, ξ is the transversal displacement in Timoshenko beam; where θ is the transversal rotation, while $\ddot{\xi }$ and $\ddot{\theta }$ are the transverse and rotary accelerations, respectively, *L* is the length of the beam, and δ denotes that the terms are virtual.

For free vibration, the stiffness and mass matrix in the element domain are written as follows:

(7) $$\begin{array}{c} \left[K^{e}\right]=\int_{-1}^{1}{\left[B\right]}^{T}\left[D\right]\left[B\right]\left|J\right|d\varsigma \\ \left[M^{e}\right]=\int_{-1}^{1}\rho I{\left[H\right]}^{T}\left[H\right]\left|J\right|d\varsigma +\int_{-1}^{1}\rho A{\left[H\right]}^{T}\left[H\right]\left|J\right|d\varsigma \end{array}$$

where [*B*], [*D*], and |J| are the strain displacement matrix, constitutive matrix, and Jacobian determinant, respectively.

(8) $$\begin{array}{c} \left[B\right]=\left[\begin{array}{cc} \begin{array}{ccc} 0 & \frac{1}{\left|J\right|}\left(\frac{d\varphi_{1}}{d\zeta }\right) & 0\\ \frac{1}{\left|J\right|}\left(\frac{d\varphi_{1}}{d\zeta }\right) & -\varphi_{1} & \frac{1}{\left|J\right|}\left(\frac{d\varphi_{2}}{d\zeta }\right) \end{array} & \begin{array}{c} \frac{1}{\left|J\right|}\left(\frac{d\varphi_{2}}{d\zeta }\right)\\ -\varphi_{2} \end{array} \end{array}\right]\\ \left[D\right]=\left[\begin{array}{cc} EI & 0\\ 0 & kGA \end{array}\right] \end{array}$$

In addition, the Block Lanczos extraction method is used in this work for its efficiency in large symmetric eigenvalue problems [7]. As the involved matrix for the finite element computation of resonant frequencies are sparse and large, the Block Lanczos extraction method is competitive when compared with other methods [28]. The Block Lanczos extraction method applied to the symmetric system generates a set of orthonormal vectors, and yields an orthogonal transformation in the generalized eigenproblem. In addition, the solver performs a Sturm sequence check at the end of the Block Lanczos calculation, which check the number of negative pivots encountered in the range that the minimum and maximum eigenvalues encompass.

### 2.3. Flowchart of Fingerprint Compiler

The geometrical configuration of graphene lattice and the corresponding geometrical and material parameters are defined as above, while the resonant frequencies and deformations under different vibration modes are computed based on the finite element method. The first four order resonant frequencies of the pristine graphene without vacancy defects are 1.7282, 3.2925, 3.7442, and 5.189 THz, respectively, which reaches good agreement with the reported literature [28,30,31].

**Figure 2:**
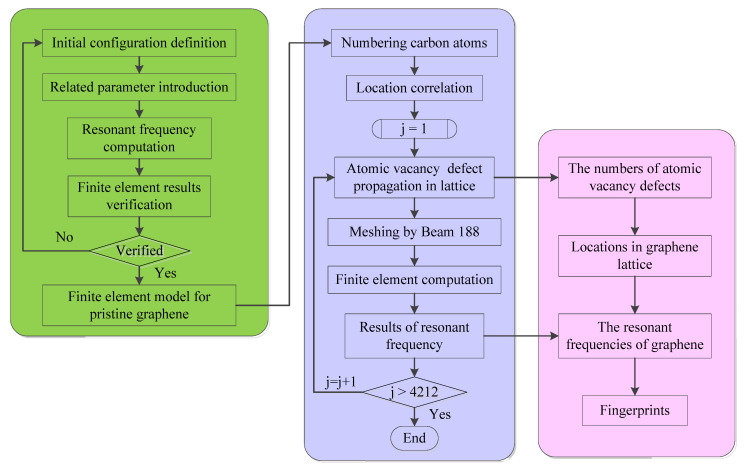
The flowchart of fingerprint compiler.

By the sequential introduction of atomic vacancy defects in the pristine perfect graphene, the relative deviations between the resonant frequencies of pristine graphene and that of vacancy-defected graphene exist. In order to analyze the impacts of atomic vacancy defects in the resonant frequencies, the numbers of vacancy-defected atoms are recorded as the input information, while the corresponding resonant frequencies of graphene are used as the output data. The flowchart of the fingerprint compiler for the relationship between the location of atomic vacancy defects and the resonant frequencies of graphene are presented in Figure 2. The implementation process of the flowchart can be concluded in the steps as follows.

Step 1: Define the initial configuration of the pristine graphene and settle the related material and geometrical parameters, which include Young’s modulus, Poisson’s ratio, mass density, cross-section diameter, and length of carbon covalent bonds, etc.

Step 2: Compute the resonant frequencies of pristine graphene by the finite element method, and also verify the finite element model for the pristine graphene.

Step 3: Number the carbon atoms in the graphene lattice and mark the location of carbon atoms with the corresponding numbers.

Step 4: Propagate the atomic vacancy defects in graphene lattice and perform finite element method for modal analysis.

Step 5: Record the location information of atomic vacancy defects and output the resonant frequencies for different resonant vibration modes, creating the database based on the huge sample space.

Step 6: Perform the atomic vacancy defect propagation and modal analysis procedure until the circles reach 4212 times. In other words, the loop recycles until all the possible situations of atomic vacancy defects are sampled and simulated.

Step 7: Picture the fingerprints of resonant frequencies according to the location distribution of atomic vacancy defects in graphene, so that the relationship between the locations of vacancy defects and resonant frequencies are visibly imaged.

In order to make the structure of the fingerprint compiler program more readable, the main framework is categorized into three components marked with different colors. The green box presents the finite element model creation procedures for pristine graphene and model verification. The violet box shows the program and recycling execution of atomic vacancy propagation. In contrast, the pink box demonstrates the last component of the fingerprint compiler, in which the database of locations of the atomic vacancy defects is mapped to the magnitudes of the resonant frequency. Then, the patterns of resonant frequency fingerprints reflect the location of atomic vacancy defects.

## 3. Results and Discussion

### 3.1. Probability Density Distribution

**Figure 3:**
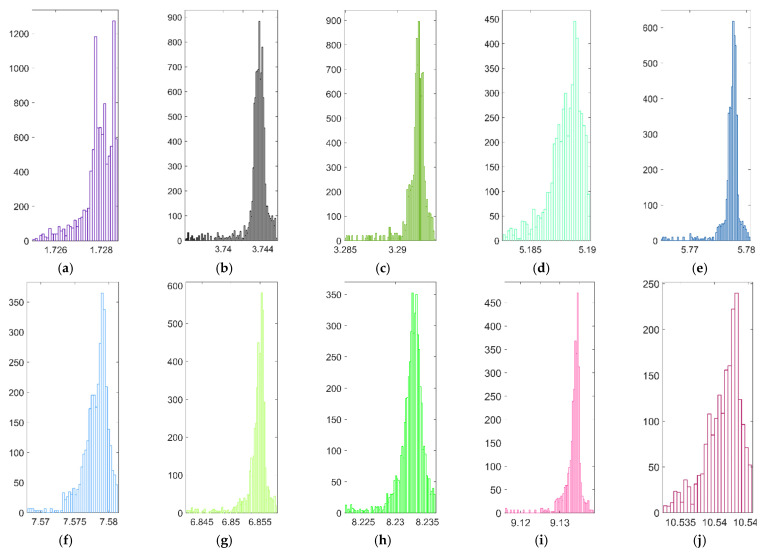

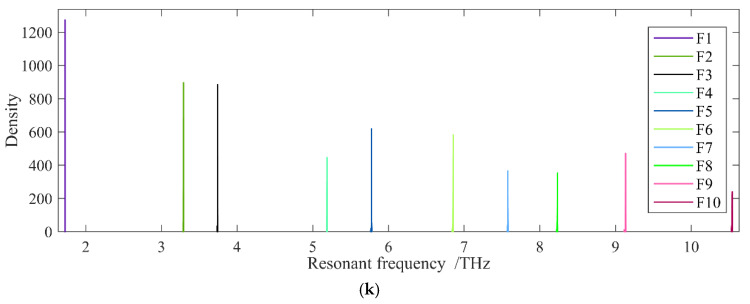
The probability density distribution of resonant frequencies (**a**–**j**) represent the first ten resonant vibration modes, respectively; (**k**) is the comparison of density in the same magnitude, and the colors in (**k**) correspond to those in (**a**–**j**), respectively.

Different from the stochastic sampling process of atomic vacancy defects propagation, all the possible locations of the atomic vacancy defects in graphene are considered and sequentially simulated. In order to analyze the impacts of atomic vacancy defects and the statistic characteristics of resonant frequencies, the probability density distributions of resonant frequencies are computed and presented, as shown in Figure 3.

From Figure 3a–j, it is obviously indicated that the sequential atomic vacancy defects cause the fluctuations and vibrations in the first ten order resonant frequencies. The probability density distribution histograms of resonant frequencies are different from the regular Gaussian, Weibull, or other analytical functions. In addition, the discrepancies in the shape of histograms and specific interval ranges reflect the complicity of atomic vacancy impacts. The impacts of atomic vacancy defects on the resonant frequencies are not completely homogeneous nor symmetric.

Figure 3k presents the probability density distributions of resonant frequencies in the same amplitude for comparison. The variations caused by the single atomic vacancy defect are not evident in the probability density distribution of resonant frequencies, and the histograms are expressed as sharp peaks. However, the differences in the peak heights reveal the discrepancies in the resonant frequencies caused by the atomic vacancy defects. The probability density distributions of resonant frequencies provide important statistical information, but the relationship between the locations of atomic vacancy defects and the responses in resonant frequencies are not explicit.

### 3.2. Contours of Fingerprints

**Figure 4:**
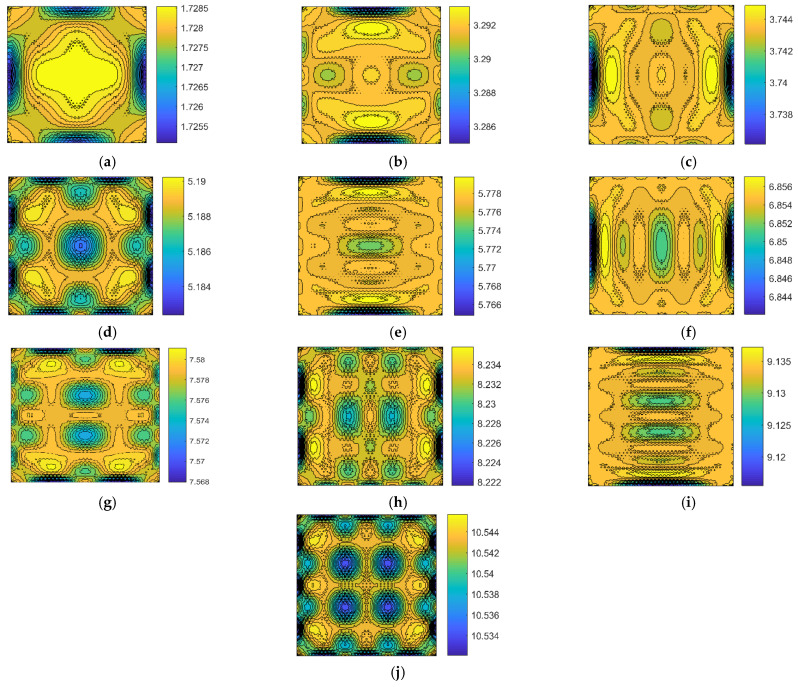
The fingerprints of the resonant frequencies pairing with the location of atomic vacancy defects in graphene ((**a**–**j**) represent the 1st to the 10th resonant vibration modes, respectively; the unit for the color bars is Thz).

The relationship between the locations of the defects and the corresponding resonant frequencies can be visualized by fingerprint patterns. In order to figure out the fingerprint patterns, the parallel data of the location of the atomic vacancy defects and the corresponding resonant frequencies of graphene are recorded and paired. The fingerprints of resonant frequencies for atomic vacancy defects in graphene are presented in Figure 4.

In the fingerprint patterns, all the first ten resonant vibration modes present geometrical symmetry. Even the values of resonant frequencies have the inhomogeneous and unsymmetrical probability density distribution, as shown in Figure 3, and the mapping of the resonant frequencies with the locations of atomic vacancy defects present strict symmetry. This phenomenon is related to the geometrical periodicity and symmetry of the honeycomb lattice. The fingerprint patterns by mapping the resonant frequencies of graphene with the locations of atomic vacancy defects present the geometrical symmetry of the periodic honeycomb lattice.

Furthermore, the contour patterns of fingerprints vary from each other for the first ten resonant vibration modes. The variances in the fingerprints are useful information for defect location identification. However, the symmetry in the patterns may bring ambiguity in the identification process since the vacancy defects located in the symmetric positions present approximated resonant frequencies. In order to predict the unknown locations of the vacancy defects by fingerprints, the symmetrical features in the patterns of fingerprints are an important issue. However, since the patterns of different fingerprints do not have the same shape and distribution, the fingerprint overlay approaches are the available methods to predict the unknown location. Therefore, the difficulties in the atomic vacancy defect identification, caused by the geometrical periodicity and symmetry of the honeycomb lattice, are foreseen, and it is necessary to discuss the identification feasibility based on the resonant frequency fingerprints.

### 3.3. Identification Feasibility

In order to discuss the feasibility of atomic vacancy identification by the fingerprints of resonant frequency, the numbers of atoms that have the approximated resonant frequencies are counted in the honeycomb lattice. In addition, relative errors are introduced to measure the identification level. Four different identification levels are settled as 10^−4^, 10^−5^, 10^−6^, and 10^−7^ Thz. At the first identification level, when the relative errors are smaller than 10^−4^ Thz, the corresponding atoms are supposed to be indistinguishable. The improvement of identification level is directly related to the distinguishable relative errors.

**Figure 5:**
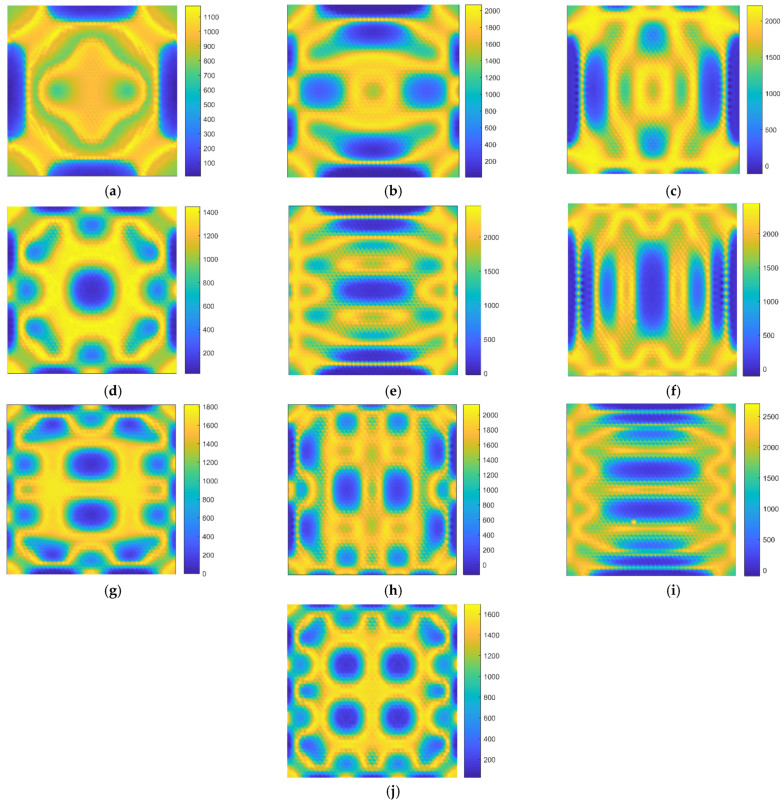
The number distributions of indistinguishable atoms with the identification limitation at 10^−4^ Thz ((**a**–**j**) represent the distributions from the 1st to the 10th resonant vibration modes, respectively).

In Figure 5, the limitation for the distinguishable relative errors is at 10^−4^ Thz. The number distributions of indistinguishable atoms present different patterns for different resonant vibration modes. According to the color bars in Figure 5, it is evident that the number of atomic vacancy defects that cause the indistinguishable resonant frequencies of graphene is as large as 2000. However, as presented in Figure 5, marked with blue color, specific atomic vacancy defects can be distinguished by the special resonant frequencies in the honeycomb lattice. Furthermore, the locations of the specific atomic vacancy defects are distributed in diverse places for the different resonant vibration modes. Therefore, identifying atomic vacancy defects by overlaying the fingerprints of different resonant vibration modes is feasible.

**Figure 6:**
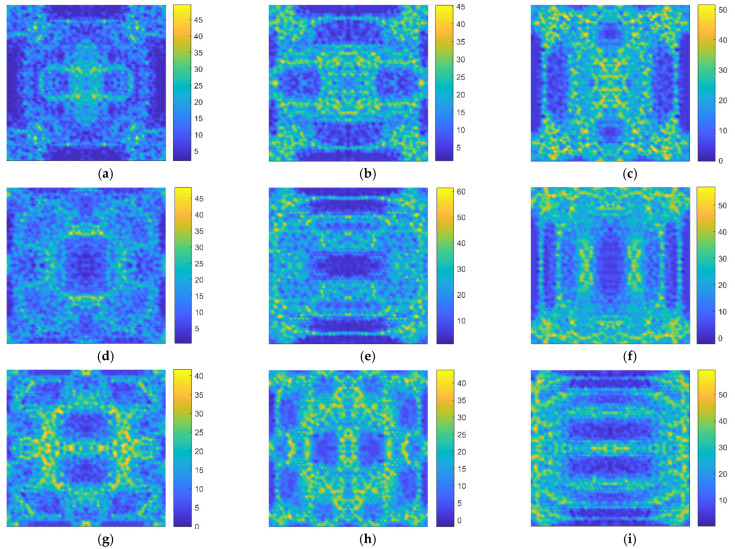

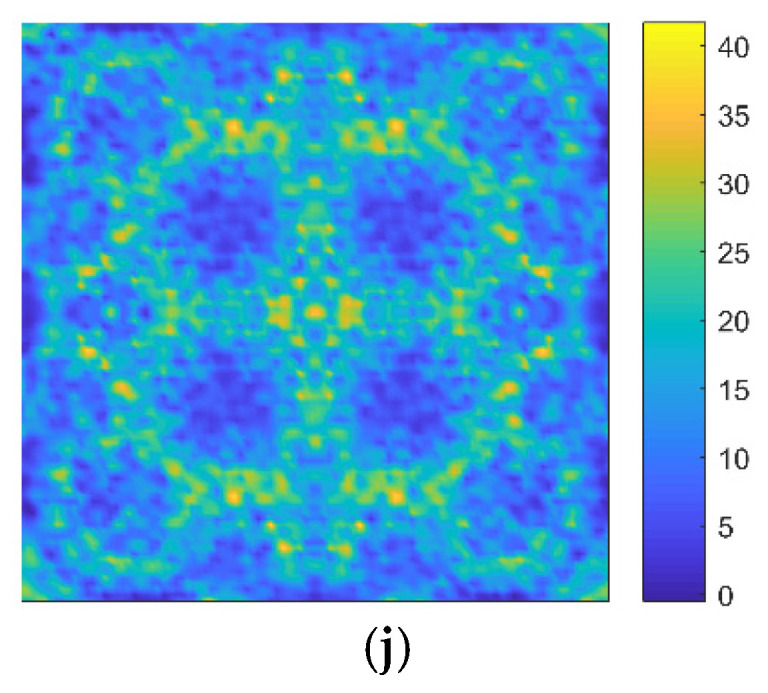
The number distributions of indistinguishable atoms with the identification limitation at 10^−6^ Thz ((**a**–**j**) represent the distributions from the 1st to the 10th resonant vibration modes, respectively).

With the more advanced identification level, the distinguishable relative error is settled as 10^−6^ Thz in Figure 6. The number of indistinguishable atoms sharply reduces with the improvement of identification level. For the same resonant vibration mode, the maximum number of indistinguishable atoms decrease evidently, from 2000 to 50. In addition, the basic patterns in Figure 6 keep the same shapes as those in Figure 4 and Figure 5 for the corresponding resonant vibration modes. The fingerprints of resonant frequencies contain fundamental information of geometrical characteristics of the honeycomb lattice of graphene, and the improvement of identification level is an efficient method for atomic vacancy identification. In addition, the fingerprints of resonant frequencies provide essential information for non-destructive identification and also act as helpful references for graphene microstructure optimization with an objective of maximizing certain material properties [32]. In addition, stress components and potential energy under shear stress in low-dimensional and heterogeneous materials are also sensitive to vacancy defects and require more concerns on defect identification and quantification [33]. In the theoretical exploration, the nonlocal elasticity theory is an essential supplement to the finite element method for the impact analysis of atomic vacancy defects [34]. The fingerprints of resonant frequencies for atomic vacancy defect identification provide important supports for non-destructive detection technology, and further work is necessary.

**Figure 7:**
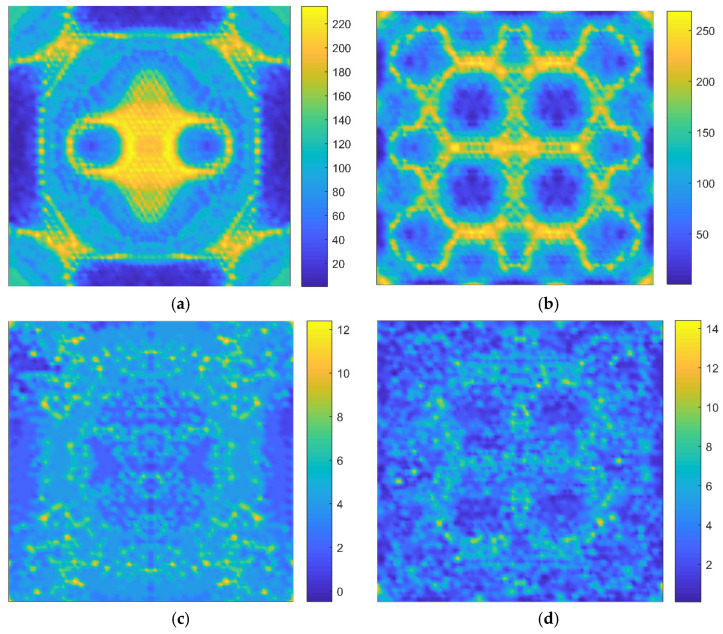
The comparison for number distributions of indistinguishable atoms ((**a**,**b**) represent the 1st and 10th resonant vibration modes with limitation at 10^−5^, while (**c**,**d**) represent those at 10^−7^, respectively).

Furthermore, the number distributions of indistinguishable atoms with different identification limitations are recorded, as shown in Figure 7. It is found that with the improvement of identification level, the number of indistinguishable atomic vacancy defects decreases, which, again, prove the results in Figure 5 and Figure 6. Moreover, the inherent characteristics, corresponding with the periodicity and symmetry of the honeycomb lattice, are proven, again, in Figure 7. Therefore, the fingerprints of resonant frequencies correlate with the inherent periodicity and symmetry of the honeycomb lattice, and the number distributions of indistinguishable atoms with different identification limitations depend on the geometrical characteristic of the lattice.

## 4. Conclusions

In this paper, the fingerprints of resonant frequencies for atomic vacancy defect identification in graphene are provided, based on finite element computation. The atomic vacancy defects are sequentially introduced and propagated in the graphene honeycomb lattice. The geometrical characteristics of the fingerprint contours and interference distribution are analyzed and discussed. In this work, the following points can be concluded.

- The impacts of atomic vacancy defects on resonant frequencies are not completely homogeneous nor symmetric.
- The fingerprint patterns produced by mapping the resonant frequencies of graphene with the locations of atomic vacancy defects present the geometrical symmetry of the periodic honeycomb lattice.
- The fingerprints of resonant frequencies contain fundamental information of geometrical characteristics of the honeycomb lattice of graphene.
- The improvement of the distinguishable level is an efficient method for atomic vacancy identification.
- The discrepancies in the fingerprints of resonant frequencies for different resonant vibration modes have the potential of overlay feasibility.
- The work in this paper provides an important reference for the identification of non-destructive atomic vacancy in graphene.

## Data Availability

Not applicable.
